# The complete chloroplast genome of *Camellia confuse* Craib 1914, an economically valuable oil crop

**DOI:** 10.1080/23802359.2022.2087547

**Published:** 2022-06-23

**Authors:** Si Wu, Mei Ying Yang, Meng Long Fan, Ying Zhang, Xin Lei Li, Heng Fu Yin, Ji Yuan Li

**Affiliations:** a Research Institute of Subtropical Forestry, Chinese Academy of Forestry, Hangzhou, China; b Nanjing Forestry University, Nanjing, P R of China

**Keywords:** *Camellia confusa*, chloroplast genome, phylogeny

## Abstract

*Camellia confuse* Craib 1914 is an industrially valuable oil crop from southern China for which little genetic information is available. Here, we found that its complete chloroplast genome is a circular sequence (156,905 bp) with a large single-copy region (LSC) of 67,724 bp, a small single copy region (SSC) of 18,400 bp, and two inverted repeats (IRs). In total, 130 genes were identified, including 86 protein-coding genes, 36 transfer RNAs, and 8 rRNA genes. Phylogenetic analysis showed that *C. confusa* is close to *C. meiocarpa*. These results provide valuable information for accelerating research on the evolution of camellias.

*Camellia confuse* Craib 1914, best known for its beautifully shaped flowers and industrially valuable oil, is a woody plant that is mainly cultivated in the Yunnan and Guizhou provinces of China, as well as in Laos, Thailand, and Vietnam (Liu et al. 2018). It belongs to the ancient genus *Camellia*, which contains about 200 species (Fan et al. 2021). Nonetheless, little genetic information for *C. confusa* is available. The chloroplast is an indispensable organelle for photosynthetic organisms and an ideal model for taxonomic classification because of its small size and conserved structure. Here, we describe the complete chloroplast genome of *C. confusa* and compare it with those of its relatives.

Leaves of *C. confusa* were sampled from the Research Institute of Subtropical Forestry, Chinese Academy of Forestry (RISF), Hangzhou, China (119°95′E, 30°07′N). All collections were approved by the head of RISF. All samples were collected by SiWu in May 10, 2021. The specimens were stored in the laboratory at the RISF, and the voucher number was YL914711 (XinLei Li, lixinlei2020@163.com). Total DNA was extracted using the MiniBest Plant Genomic DNA Extraction Kit (Takara, Dalian, China), and sequenced on the Illumina HiSeq 4000 platform (Illumina, San Diego, California, USA) at Genesky Biotechnologies (Shanghai, China). We obtained a total of 25,129,048 reads, and 23,938,766 clean reads remained after quality control with Trimmomatic (Bolger et al. 2014). SPAdes version 3.10.1 (Bankevich et al. 2012) was used to assemble the chloroplast genome, and Velvet Optimizer version 2.2.5 was used to maximize the splicing results. We used CpGAVAS2 (Shi et al. 2019) to annotate the final genome. BLASTp at NCBI was used to confirm the annotation of the protein-coding sequences. Information on the chloroplast genome was uploaded to NCBI (MW034673).

The chloroplast genome of *C. confusa* was 156,905 bp in length with a GC content of 37.31% and showed the typical quadripartite structure, with a large single-copy (LSC) region of 67,724 bp, a small single copy (SSC) region of 18,400 bp, and two inverted repeat (IR) regions of 24,662 bp. Overall, it contained 86 protein-coding genes, 36 transfer RNAs, and 8 ribosomal RNAs. Among these genes, 15 (atpF, ndhA, ndhB, petB, petD, rpl16, rpl2, rpoC1, rps12, rps16, trnA-UGC, trnG-GCC, trnI-GAU, trnK-UUU, trnL-UAA, and trnV-UAC) contained one intron, and two genes (clpP and ycf3) contained two introns.

To investigate the evolutionary relationships of *C. confusa* with other members of the genus, we performed phylogenetic analysis of thirteen *Camellia* species. MEGA v7.0.14 was used to construct the phylogenetic tree by the maximum likelihood method (Kumar et al. 2016). The result revealed that *C. confusa* was closely related to *C. meiocarpa* (Figure 1).

**Figure 1.**
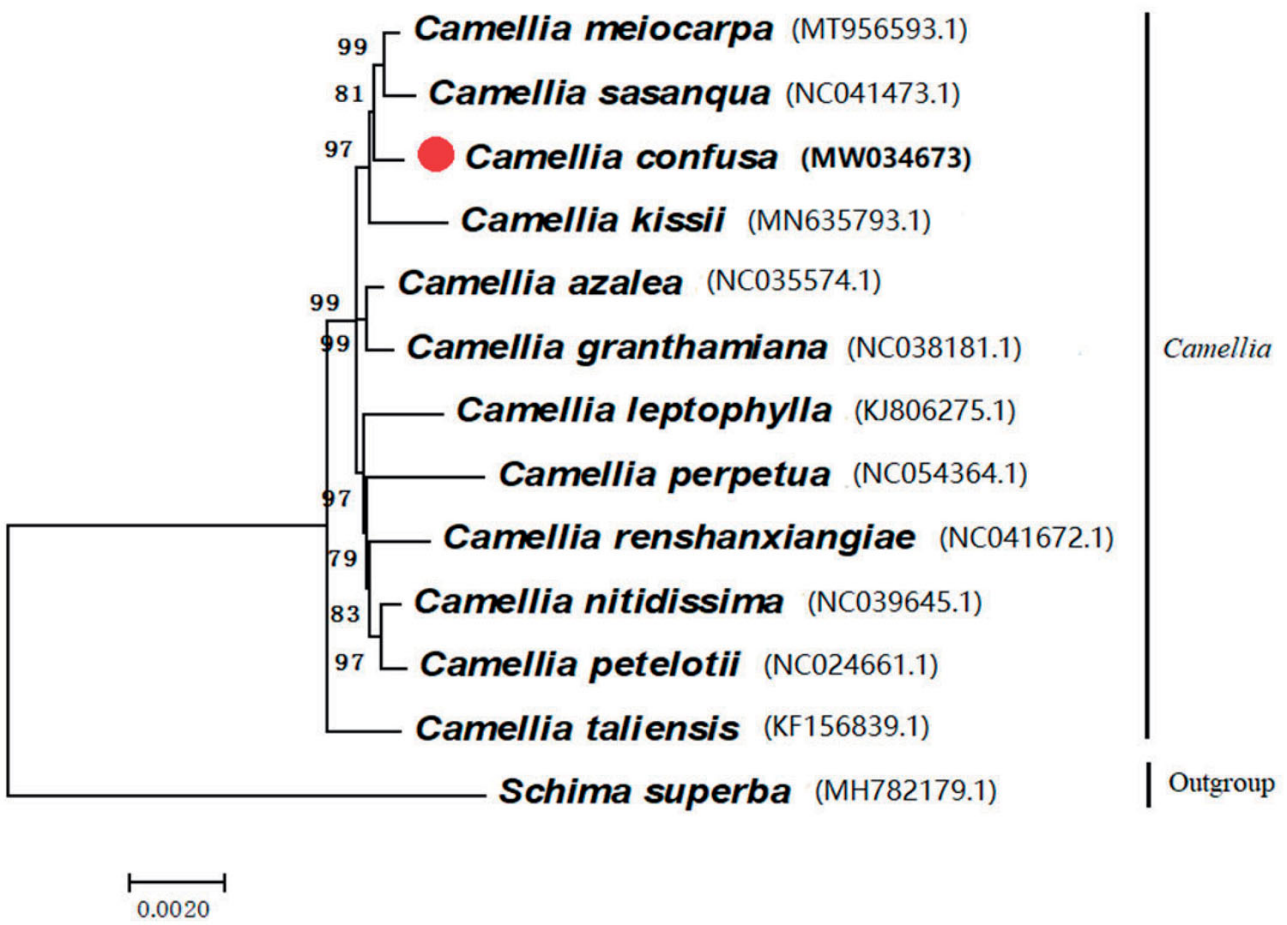
Maximum likelihood phylogenetic tree based on protein-coding genes of *C. confusa* and other *Camellia* species, with *Schima superba* as an outgroup.

## Author contributions

SiWu drafted the manuscript; Meiying Yang and Menglong Fan performed the data analysis; Ying Zhang and Xinlei Li designed this study and revised the manuscript critically for intellectual content; Hengfu Yin and Jiyuan Li carried out literature search, data acquisition and manuscript editing; all authors contributed to the final approval of the version to be published and all agree to be accountable for all aspects of the work.

## Supplementary Material

Supplemental Material

## Data Availability

The genome sequence data that support the findings of this study are openly available in GenBank of NCBI at [https://www.ncbi.nlm.nih.gov] under the accession No.MW034673. The associated BioProject, SRA, and Bio-Sample numbers are PRJNA745398, SRR15097284, and SAMN20165932 respectively.

## References

[CIT0001] Bankevich A, Nurk S, Antipov D, Gurevich AA, Dvorkin M, Kulikov AS, Lesin VM, Nikolenko SI, Pham S, Prjibelski AD, et al. 2012. SPAdes: a new genome assembly algorithm and its applications to single-cell sequencing. J Comput Biol. 19(5):455–477. 22506599 10.1089/cmb.2012.0021 PMC3342519

[CIT0002] Bolger AM, Lohse M, Usadel B. 2014. Trimmomatic: a flexible trimmer for Illumina sequence data. Bioinformatics. 30(15):2114–2120. 24695404 10.1093/bioinformatics/btu170 PMC4103590

[CIT0003] Fan ML, Yang K, Zhou R, Liu QH, Guo X, Sun YK. 2021. Temporal transcriptome profiling reveals candidate genes involved in cold acclimation of Camellia japonica (Naidong). Plant Physiol Biochem. 167:795–805. 34530324 10.1016/j.plaphy.2021.09.006

[CIT0004] Kumar S, Stecher G, Tamura K. 2016. MEGA7: molecular evolutionary genetics analysis version 7.0 for bigger datasets. Mol Biol Evol. 33(7):1870–1874. 27004904 10.1093/molbev/msw054 PMC8210823

[CIT0005] Liu C, Longsheng C, Wei T, Shaofeng P, Meiqun L, Nan D, Yongzhong C. 2018. Predicting potential distribution and evaluating suitable soil condition of oil tea *Camellia* in China. Forests. 9(8):487.

[CIT0007] Shi L, Chen H, Jiang M, Wang L, Wu X, Huang L, Liu C. 2019. CPGAVAS2, an integrated plastome sequence annotator and analyzer. Nucleic Acids Res. 47(W1):W65–W73. 31066451 10.1093/nar/gkz345 PMC6602467

