# Supplementary material for: The complete chloroplast genome of Camellia confuse Craib 1914, an economically valuable oil crop
Source: Mitochondrial DNA B Resour. 2022 Jun 23;7(6):1099–100. doi: 10.1080/23802359.2022.2087547 (PMC9246039; doi:10.1080/23802359.2022.2087547)
Supplement: Supplemental Material [file TMDN_A_2087547_SM1962.pdf]

EditorBar
北京艾迪润色

EditorBar Language Editing
No. 35, Tsinghua East Road, Beijing, China 100083
Email: runse@editorbar.com Phone: +86-10-5620-8614

CERTIFICATE OF LANGUAGE EDITING

The English writing of the following manuscript was carefully edited by a native English speaker.

Manuscript Information

ID	LE202203290673
Editing date	2022-04-02
Title	The complete chloroplast genome of Camellia confusa, an oil crop with economic value
Corresponding author	xinlei li
Language writing before editing	□ Very poor □ Poor □ Fair ■ Good □ Very good □ Excellent
Recommendation after language editing	■ Submitting to target journal directly<br>□ Submitting to target journal after minor revision<br>□ Re-editing required after major revision<br>□ Not suitable for publication

Overview comments	This manuscript has been thoroughly edited for English word choice, grammar, and sentence structure. Thank you for choosing me as your Editor Bar editor, and best wishes for continued research success!

Edited by

Christina E.W.

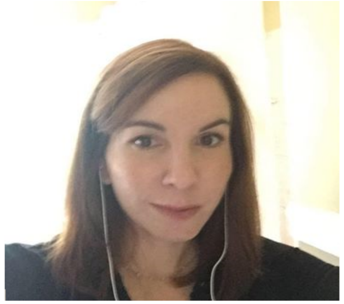

Senior Editor

The Pennsylvania State University

Language Editing

Certificate Issued by

Dr. Jason Qee

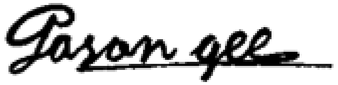

Editor in Chief

Editorbar Language Editing, Beijing, China

runse@editorbar.com   www.editorbar.com

Certificate link: www.editorbar.com/order/cert/LE202203290673
